# Land use and land cover changes and their impact on ecosystem service values in the north-eastern highlands of Ethiopia

**DOI:** 10.1371/journal.pone.0289962

**Published:** 2023-09-08

**Authors:** Meseret Muche, Getahun Yemata, Eyayu Molla, Wubetie Adnew, A. Muthama Muasya

**Affiliations:** 1 Department of Biology, College of Science, Bahir Dar University, Bahir Dar, Ethiopia; 2 Department of Biology, Woldia University, Woldia, Ethiopia; 3 Department of Natural Resource Management, College of Agriculture and Environmental Sciences, Bahir Dar University, Bahir Dar, Ethiopia; 4 Department of Biological Sciences, University of Cape Town, Rondebosch, Cape Town, South Africa; Van Lang University: Truong Dai hoc Van Lang, VIET NAM

## Abstract

The land use and land cover (LULC) changes driven by the growing demands of mankind have a considerable effect on ecosystem services and functions. The study was carried out in the north-eastern highlands of Ethiopia to (1) analyze the effect of LULC changes between 1984 and 2021 and (2) assess the spatiotemporal variations in ecosystem service values (ESVs) and elasticity in response to LULC changes. Using Landsat imageries from 1984 to 2021, the spatiotemporal changes in LULC were evaluated with supervised image classification using maximum likelihood algorithm in ArcGIS software. Six LULC types were subsequently categorized, with overall accuracy and Kappa coefficients above 87% and 0.87, respectively. The ESVs were then estimated based on the Benefit Value Transfer (BVT) approach employing modified conservative value coefficients. The findings revealed a significant increase in cultivated land (9759.1ha) and built-up area (10174.41ha) during the stipulated periods and a drop in other land use types. The forest loss gradually decreased from 4.1% in the second period (1991–2001) to 0.58% in the third (2001–2021), compared to the first of the 1.1% conversion rates. Similarly, the proportion of grassland and water bodies steadily reduced over the stipulated periods, by 1.15% and 2.3% per annum, respectively. The overall loss of ESVs in the study landscape was estimated to be 54.4 million US$ (67.3%), drastically decreasing from 80.3 million US$ in 1984 to 26.4 million US$ in 2021, driven by the declining area coverage of water bodies, grassland, and forestland. Regardless of the loss, the ecosystem functions of hydrological regulation (37.2, 35.0, 6.1, and 5.1 US$ ha^-1^yr^-1^), water supply (14.5, 13.6, 2.4, and 2 US$ ha^-1^yr^-1^), and food production (9.8, 10.0, 9.1, and 9.9 US$ ha^-1^yr^-1^) contributed the most to the total ESV of each year while disturbance regulation and cultural values were the least throughout the study periods. The coefficient of sensitivity (CS) analysis revealed that our estimates were relatively robust. The findings further showed that human-dominated land-uses at the expense of natural ecosystems are the primary drivers of LULC transitions and the ensuing loss of ecosystem services in the region. Thus, this calls for intensive work on more effective land use policies that encourage an integrated management approach, with a focus on safeguarding the sustainability of natural ecosystems.

## Introduction

Tropical forest ecosystems provide a wide range of services including habitat conservation, water and soil conservation, biodiversity preservation, and carbon sequestration [1, 2]. However, nearly half of the tropical forest that existed at the beginning of the century has already vanished [3]. Land-use change is thought to be the major driver of the loss of forest ecosystems and the change in the accompanying services [1, 4]. Changes in land use/land cover (LULC) have a marked effect on local-to-global ecological processes and ecosystem services [2, 4], pose a serious threat to biological resources, such as forests, grasslands, wetlands, and other ecosystems [1, 5], and have a profound impact on achieving Sustainable Development Goals (SDGs) [5, 6]. Collectively, all of which have a strong influence on carbon sources and sinks as well as global environmental change. The main driving forces of the LULC transition might be connected to multiple factors, such as the increasing human population and accompanying consumption demands [7, 8], rapid socio-economic development [9, 10], policies and institutional factors [10, 11]. Climate-induced changes (e.g., drought, precipitation variability, forest fire, invasive species, and other disturbances) can also put pressure on LULC alterations [2, 12, 13].

In Ethiopia, LULC changes especially forest cover transition has been more severe in the highland areas (altitude >1500 m) over the past few decades [14–21], as a result of increasing population, which led to significant forest clearance for agricultural use, overgrazing, etc. The forest under this threat covers more than half of the Afromontane forests of the country [14], which is one of the key biodiversity hotspots in Eastern Africa with a diverse flora and significant ecological reservoirs [12, 22]. According to FAO [23], Ethiopia’s forest cover decreased from 15.11 million ha in 1990 to 12.5 million ha in 2015. Several recent studies have also shown that the proportion of forested land reduced by 8.2% in the Northern [24], 3.5% in the North-western [17], 34.45% in the Western [25], over 45% in the south western [26], and 13% in the central highlands of Ethiopia [21]. A substantial decline in forest cover has been attributed to its conversion to abandoned pasture and croplands. In most cases, immense pressures from anthropogenic activities (e.g., poor agricultural practices, deforestation, overgrazing, etc.) coupled with a rapidly increasing population are the main drivers for changes [14–18, 24, 25, 27]. As a result, forest resources, mainly natural forests, are increasingly becoming scarce [14, 22], resulting in habitat fragmentation, biodiversity loss, and a reduction in ecosystem services.

Ecosystem services refer to the benefits that humans derive from natural ecosystems and their components, including provisioning services (e.g., food, timber, fodder, water, medicinal products), regulating services (e.g., carbon storage and sequestration, water purification, preventing soil erosion), supporting services (e.g., habitat, biodiversity conservation), and cultural services (e.g., aesthetic, recreational, and tourism) [5–8]. Human well-being and the functioning of the global economy depend on ecosystem services, but these services are under threat because of the intricate interplays between people and the environment, which result in ecosystem degradation and biodiversity loss [28], and lead to economic impoverishment. It is estimated that 60% of ecosystem services have changed in just the last 50 years [5, 6], with human-caused LULC alterations being the primary causes [5–8]. Even though ESVs vary spatially, Costanza et al. [8] estimated that global LULC changes have caused ESVs to decrease from US$ 145 trillion year^−1^ in 2007 to US$ 125 trillion year^−1^ in 2011. The decline in ESVs is more pronounced and burgeoning in developing countries like Ethiopia [29–31]. Recent studies in Ethiopia [29, 31, 32] have asserted that the changes in ESVs resulting from LULC changes have led to a significant reduction in forest coverage and an increase in croplands. According to Tolessa et al. [30], forest conversion has resulted in a loss of 40.7% (3.69 million) in ESVs in the central highlands of Ethiopia, and comparable results have been found in other parts of the country [32, 33].

To avert the scenarios of forest degradation and ESVs loss, Ethiopia’s government has adopted the Green Legacy Initiative through sustainable forest management and restoration programs, which aims to address the issue of forest degradation and loss of ecosystem services through sustainable forest management and restoration programs [34]. These initiatives are designed to monitor and manage regional ecosystem changes, provide a framework for ecological restoration and conservation, and also form sustainable development policies, with a focus on the highlands [34]. The North-eastern highlands of Ethiopia are notable for having mosaics of different LULC types where dry evergreen Afromontane forests have historically been degraded as a result of human pressure. However, there have been recent efforts by the government to restore these forests, including community-driven initiatives such as tree planting, enclosures, and exclosures, as well as soil and water conservation measures (SWC) for over two decades. Despite these interventions, previous research has not investigated the impacts of LULC changes on ESVs in the region, considering the differences in landscapes and restoration endeavours. Therefore, this study was initiated to analyse the (1) trends in LULC changes/trajectories and vegetation covers of the area from 1984 to 2021, (2) spatiotemporal variation in ESVs of the area caused by LULC change in the stipulated periods, and (3) contribution of individual ecosystem service values and elasticity driven by landscape dynamics during the study periods. The findings of the study were instrumental to show the environmental and ecological changes, project additional intervention options and provide evidence-based decisions concerning regional sustainable development.

## Materials and methods

### Description of the study area

**Fig 1:**
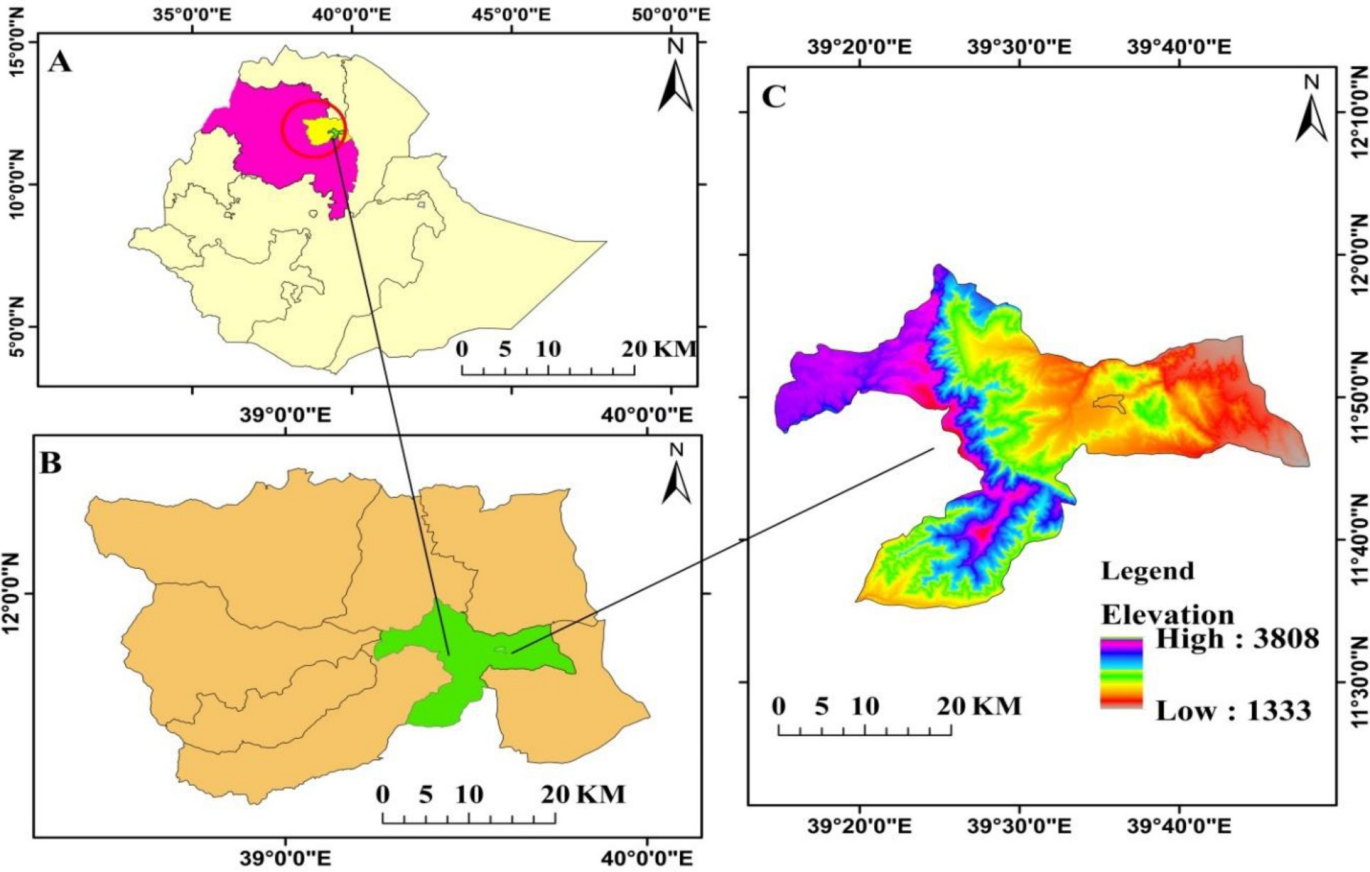
Map of the study areas (ArcGIS version 10.7.1 software, Esri, California, USA). The illustration shows (A) location of Amhara National Regional State, (B) North Worth Wollo by districts, and (C) Study areas (Woldia city administrative and Guba Lafto district).

The study area falls into two districts, namely Guba Lafto and Woldia administrative city. The areas are part of the North Wollo administrative zone of the Amhara National Regional State (ANRS), in the Northern eastern Ethiopian highlands (Fig 1), and are located between 11°20’00’’N to 12°10’.00"N latitude and 39°20’00’’ to 39° 40’ 40.00" E longitude. The study area is part of the north-central plateau, consisting of a chain of mountains, hills, valleys, plains, and plateaus with topography that ranges from nearly 1379 to 3200 meters above sea level (m.a.s.l) [35]. The study area has predominantly midland (*Woina Dega*) and highland (*Dega*) agro-ecological zones, with a bimodal rainfall pattern (from February to March and from June to August). It has an average annual rainfall ranging from 10 mm in January to 380 mm in August and temperatures of 12.4 to 28.7°C [36, 37]. The major soil types of the area are the vertisols, nitosols, and cambisols [38]. The main economic activities are farming and livestock rearing, and the dominant crops are *Sorghum bicolor*, *Zea mays*, *Eragrostis tef*, and *Cicer arietinum*, but the area is often highly degraded. Despite centuries of human use and land modification, the natural vegetation of the study area belongs to the remnant dry evergreen Afromontane forests of the Ethiopian highlands [39]. Natural forests can be found on steep slopes and in churchyards, dominated by *Olea europaea subsp*., *cuspidata*, *Eucalyptus camaldulensis*, *Acacia spp*, etc.

### Data acquisition

Changes in land use/land cover (LULC) are the major factors affecting ecosystem processes and services [29]. The changes are happening at a fast rate due to an increase in human demands on the forest ecosystem [40]. Thus, the evaluation of long-term regional and global LULC change is made by the Landsat data archives, which are imperative for historical records of the Earth’s land surface [41]. For this study, multispectral satellite imageries were downloaded from the United States Geological Survey Earth Explorer website (USGS) (https://earthexplorer.usgs.gov/) to analyse LULC changes in the study area over 37 years (Tables 1 and 2). Hence, Landsat 4–5 Thematic Mapper (TM) of 1984, Landsat5 Thematic Mapper (TM) of 1991, Landsat7 Enhanced Thematic Mapper Plus (ETM+) of 2001, and the Landsat8 Operational Land Imager (OLI) of 2021 were used for this study (Table 1). The study was purposely commenced in 1984, as it aligned with a time when deforestation was rampant, particularly in the dry evergreen Afromontane regions of the country [42]. It is crucial to ascertain the magnitude of the issue in the region and devise a proactive decision-making process towards solving intricate environmental problems. The images were captured with high-resolution (30m x 30m) during cloud-free scenes of the dry season (December to January) to avoid the effects of seasonal variations in ground cover especially effects of annual crops or cloud cover, which might affect the data interpretation [17, 41, 43], and are particularly valuable for studying natural and agricultural environments, as they can enable informed decision-making that promotes sustainable land use and healthy plant communities [17, 43]. These images were projected to the Universal Transverse Mercator (UTM) using the World Geodetic System (WGS) 84 zone 37 North datum. In addition, auxiliary data (ground truth points) were acquired between September and October of 2022 using a portable handheld Global Positioning System (GPS) and a Google Earth satellite image for the study area. Hence, a total of 350 GPS points (50 points per class) were used to classify and validate LULC classifications.

**Table 1:** Description of satellite images used for the study.

Sensor	Imagery type	Acquisition date	Pixel size (m)	Path/Row	Source
TM	Landsat 4–5	1984/12/08	30*30M	168/52	USGS
TM	Landsat 5	1991/01/19	30*30m	168/52	USGS
ETM+	Landsat 7	2001/01/22	30*30m	168/52	USGS
OLI	Landsat 8	2021/01/05	30*30m	168/52	USGS

**Table 2:** LULC classes and their thematic description in the study area.

LULC class	Descriptions
Forestland (dry evergreen Afromontane forest)	Land with more than 0.5 ha, covered with a tree canopy of more than 10%, which was not primarily under cultivation or other specific non-forest land uses.
Cultivated land	Areas of land ploughed/prepared for growing crops by rainfed or irrigation, including fallow plots.
Grassland	Areas utilized for communal grazing or dominated by grass, and it includes areas covered with natural grass and tiny bushes.
Built-up area	Areas occupied by residential houses and other buildings.
Barren land	Dry and bare landscape dominated by rocks, outcrops and cliff, and has very few plants and no trees
Water bodies	Areas of land covered with water (lakes and rivers).

### Image pre-processing and analytical methods

All imageries underwent pre-processing (e.g., layer stacking, mosaic, geometric and radiometric calibration, and panchromatic) using ArcGIS (version 10.7.1 software, Esri, California, USA) to enhance the image quality. The radiometric calibration was carried out using the raster calculator (Spatial Analyst) tool. However, the geometric adjustment was not required because the pixels in the Landsat images from the USGS website are accurate [44].

### Normalized Difference Vegetation Index (NDVI)

The Normalized Difference Vegetation Index (NDVI) is one of the most often used metrics for detecting vegetation conditions (e.g., cover, biomass, primary production, and carbon balance) [44–46]. It also allows for the evaluation of spatial and temporal changes occurring on the Earth’s surface using reflectance measurements [45, 47]. In this study, NDVI values were calculated based on the reflectance of the red (R) and near-infrared (NIR) bands (Eq 1) of satellite images (https://earthexplorer.usgs.gov/).


$$NDVI=\frac{\left(NIR-R\right)}{\left(NIR+R\right)}$$ (1)

The NDVI ranges from -1.0 to 1.0 [45]. According to Hartoyo et al. [48], NDVI values are categorised into five classes, namely class 1 (non-vegetation areas or water bodies, -1 to -0.03), class 2 (very low vegetation, -0.03 to 0.15), class 3 (low vegetation, 0.15 to 0.25), grassland (moderately vegetation, 0.25 to 0.35), and class 5 (thriving and very dense vegetation, 0.35 to 1).

### Supervised images classification

Supervised image classification was employed using the maximum likelihood algorithm, which is a widely used technique in image classification [49]. Ground truth points from each LULC category were collected to aid in the classification process. The supervised classification method is commonly used to identify land use classifications using spectral signatures [50], and it assumes that the statistics are normally distributed for each class and in each band [51]. The image was then sorted into groups based on how closely it resembled the training signatures after collecting samples from the various land cover classes (training data) [52]. The variance and covariance of the spectral response patterns were quantitatively evaluated using the maximum likelihood algorithm, and each pixel was then assigned to the class for which it has the greatest likelihood of association [41]. The LULC classes that were identified are forestland, cultivated land, grassland, built-up areas, barren land, and water bodies (Table 2).

### Detection of land use/land cover changes

A post-classification approach was utilized for identifying LULC changes over time based on the numerical values extracted from classified images [41, 44, 51, 53]. This technique involves overlay comparisons to determine which LULC types have been converted [41, 54]. To do this, classification images of the same region generated independently at distinct time periods are compared. Then, the final classified thematic maps from subsequent periods (i.e., 1984–1991, 1991–2001, 2001–2021, and 1984–2021) were cross-tabulated [29, 33, 41, 44] to determine the percentage and rate of land use/land cover change detections over time series. To further understand the observed LULC transitions, the total and net changes of the area were calculated using Eq 2.


$${LULC}_{I}=\frac{{LULC}_{end\;year}-{LULC}_{start\;year}}{{LULC}_{start\;year}}*\frac{1}{t}*100$$ (2)

Where, *LULC*_*I*_ is the degree to which LULC changed or transformed during the reference period *i*, *LULC*_*end year*_ and *LULC*_*start year*_ represent the area of a particular land use unit at their respective beginning and ending times, and t denotes the estimation period. Thus, positive values indicate an increase in the extent of LULC, whereas negative values show a decrease.

### Accuracy assessment

The accuracy assessment indicates the degree to which a particular land cover on the ground deviates from the reference map or the frequency with which actual features on the ground are accurately depicted on the classified map [41, 55]. All of the classified imageries in the current study had their accuracy evaluated using ground truth points collected from varied land use types in the eco-region via: (1) direct data acquired using GPS (Garmin GPSMAP 60CSx) from the field, (2) information obtained from interviews with elders and communities in the study area (3) Woody Biomass Inventory and Strategic Planning Project data (https://www.acronymfinder.com/Woody-Biomass-Inventory-and-Strategic-Planning-Project-(Ethiopia)-(WBISPP).html), and (4) Google Earth data sets. As a result, the reliability of the classified LULC was assessed using confusion matrices: the user’s accuracy, producer’s accuracy, overall accuracy (Eq 3) [51] and kappa coefficient (Eq 4) [56] as follows

$$Overall\;accuracy=\frac{\sum_{i=1}^{r}Xii}{X}$$ (3)

Where, Xii is the diagonal elements in the confusion matrix, X is the total number of samples in confusion matrix

$$K=\frac{M\sum_{i=j=1}^{r}nij-\sum_{i=j=1}^{r}ninj}{M^{2}-\sum_{i=j=1}^{r}ninj}$$ (4)

Where, K = kappa statistics, M = total number of observations in the matrix, r = number of rows in the confusion matrix, n_ij_ = number of observations in row i, column j, n_i_ = total number of observations in row I, n_j_ = total number of observations in column j. According to Viera and Garrett [56], values of kappa statistics range from -1 to 1, i.e., values greater than 0.80 denote substantial or strong agreement, values between 0.40 and 0.80 represent moderate agreement, and less than 0.40 signify poor agreement between classification and reference data.

### Estimation of ecosystem service values (ESVs)

Ecosystem service (ES) valuation signifies the monetary value assigned to a variety of ecosystem services offered by the natural environment [8, 57, 58]. This value is predicated on the assumption that the average unit value across all locations is homogeneous, which may not be the case in reality [8, 29, 58]. In this study, the benefit value transfer (BVT) approach was employed to estimate the ecosystem service values (ESVs) in response to the spatiotemporal LULC change of different land uses for the years 1984, 1991, 2001, and 2021. The BVT allows estimates for one site to be based on estimates from other sites or data from studies that have previously been conducted in different places [29–33, 59, 60]. Costanza et al. [56] have classified the whole biosphere into 16 biomes and 17 service function types (Tables 3 and 4). The ESVs of each LULC types at various locations are estimated, and thus, the equivalent value per unit area of ES for the study area was extracted using the modified conservative value coefficients, based on data from Kindu et al. [29], which was modified after Costanza et al. [8, 57] models. This is because the study area has comparable similar market values and geographical settings, notably tropical Afromontane region (e.g., 1500–3000 m.a.s.l). Accordingly, ESV per unit area for each LULC category (Table 2) was assigned based on the corresponding coefficient value of the ES (Tables 3 and 4). Land use classifications such as barren land and settlement did not have a coefficient in other studies [e.g., 8, 29–33, 57, 58]. Similarly, no coefficients were assigned to built-up areas and barren land in this study since the LULC classes lacked vegetation. The ecosystem service value (Eq 5), ecosystem function (Eq 6), and total ESV (Eq 7) for each thematic class were computed after evaluating the ESV per unit area for each land cover class using the models described by Costanza et al. [8, 57] and recommended by Kreuter et al. [61] and Kindu et al. [29].


$${ESV}_{k}=\sum_{f}A_{k}*{VC}_{kf}$$ (5)

$${ESV}_{f}=\sum_{k}A_{k}*{VC}_{kf}$$ (6)

$$ESV=\sum_{k}\sum_{f}A_{k}*{VC}_{kf}$$ (7)

Where, ESV_k_, ESV_f_, and ESV refer to the ecosystem service value of LULC type `k’, service function f, and the total ESVs, respectively (in unit of US$). A_k_ represents the area (ha) for LULC category `k’ and *VC*_*kf*_ is the value coefficient for land use type k with ecological service function type f.

The changes in ESVs were evaluated by calculating the difference between the estimated values in each reference year (Eq 8).


$$ESV\;(\%)=\frac{{ESV}_{final\;year}-{ESV}_{initial\;year}}{{ESV}_{inital\;year}}*100$$ (8)

**Table 3:** Summary of land use/land cover (LULC) types and their equivalent biomes with the corresponding value coefficients (US$ ha^−1^ year^−1^) based on the modified conservative value coefficients adopted from Kindu et al. [2,9].

Land use land cover types	Equivalent biomes	US$ (ha^−1^ year^−1^)
Forestland (DAFs)	Tropical forest	986.69
Cultivated land	Croplands	225.56
Grassland	Grass/rangelands	293.25
Built-up area	Urban	0
Barren land	Desert	0
Water bodies	Lakes/rivers	8103.5

**Table 4:** Ecosystem service values of functions of each LULC type based on the Economic Ecosystem and Biodiversity valuation database [29].

Functional types of ecosystem services	Accounting index	LULC types ecosystem service values (US$/ha/yr)					
		Forest DAFs	Cultivated land	Built-up Area	Grassland	Barren lands	Water bodies
Provisioning services	WS	8					2117
	FP	32	187.56		117.45		41
	RM	51.24					
	GR	41					
Regulatory services	Gr	13.68			7		
	Cr	223					
	Dr	5					
	Hr	6			3		5445
	EC	245			29		
	BC		24		23		
	WT	136			87		431.5
Supporting services	NC	184.4					
	SF	10			1		
	Ha	17.3					
	Po	7.27	14		25		
Recreation and culture	Re	4.8			0.8		69
	Cu	2					
**Total**		**986.69**	**225.56**	**0**	**293.25**	**0**	**8103.5**

**Notes**: WS, Water Supply; FP, Food Production; RM, Raw Materials; GR, Genetic Resources; Gr, Gas regulation; Cr, Climate regulation; Dr, Disturbance regulation; Hr, Hydrological regulation; EC, Erosion Control; BC, Biological Control; WT, Waste Treatment; NS, Nutrient Cycling; SF, Soil Formation; Ha, Habitats; Po, Pollination; Re, Recreation; Cu, Cultural

### Analysis of coefficient of sensitivity

The sensitivity analysis was used to verify the ESV variations in response to ±50% adjustments of the ESV coefficients for each type of LULC [29, 62]. The coefficient of sensitivity (CS) was therefore determined based on the standard economic concept of elasticity of formula noted by Kreuter et al. [61]. It follows as:

$$CS=\frac{({ESV}_{j}-{ESV}_{i})/{ESV}_{i}}{({VC}_{jk}-{VC}_{ik})/{VC}_{ik}}$$ (9)

Where, ESV_i_ and ESV_j_ are the initial and adjusted total estimated ecosystem service values, respectively. VC_ik_ and VC_jk_ are the initial and adjusted value coefficients (US$ ha^−1^ year^−1^) for LULC type ‘k’. The estimated ecosystem service value is, however, elastic for the value coefficient if the changes of the two values are greater than the threshold limit (> 1), and it is fully inelastic when the value is less than one [61].

## Results

### Accuracy assessment of LULC maps

Table 5 displays the accuracy assessment for the identified LULC types. For the years 1984, 1991, 2001, and 2021, the overall categorization accuracy ranges from 87 to 91%. According to Monserud and Leemans [63], a very good kappa value should fall between 0.70 and 0.85. In this study, the classified images exhibited a kappa coefficient above 0.80. As a result, the validation data set revealed a very strong agreement between the categorized images and the ground truth data.

**Table 5:** Accuracy assessment of LULC for 1984, 1991, 2001, and 2021.

LULC	**1984**		**1991**		**2001**		**2021**	
	Producer’s	User’s	Producer’s	User’s	Producer’s	User’s	Producer’s	User’s
Cultivated land	89	90	81	88	82	86	85	87
Barren land	87	82	90	84	88	83	95	92
Built-up area	89	89	89	85	90	93	100	90
Forestland	88	90	92	90	90	88	93	100
Grassland	87	88	90	91	88	93	92	89
Water bodies	89	88	93	92	89	90	100	100
Overall accuracy	88		89		87		91	
Kappa statistics	0.86		0.88		0.85		0.90	

### Land use land cover (LULC) changes

#### Trends of LULC trajectories, 1984 to 2021

**Fig 2:**
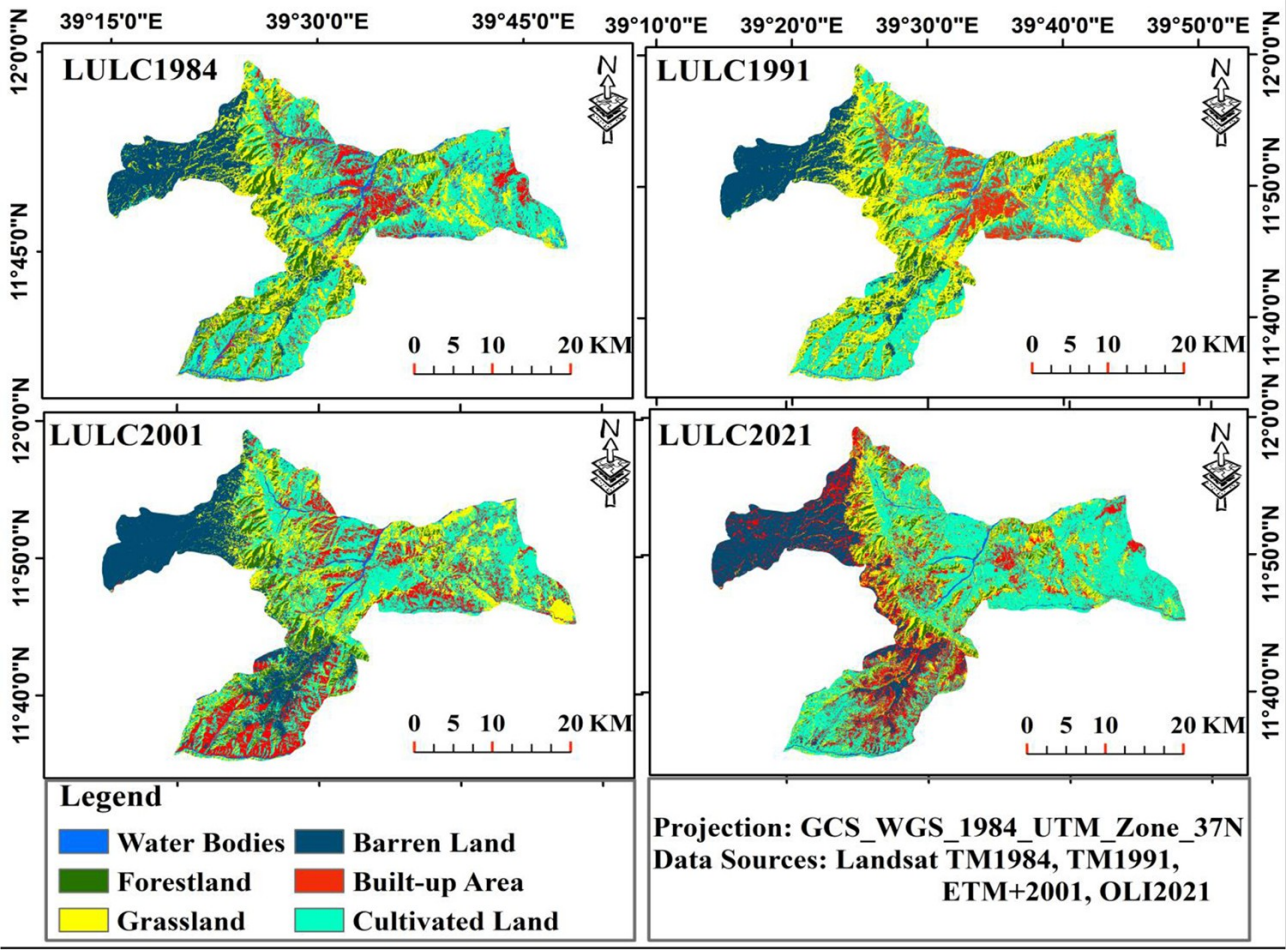
Land use/land cover change for stipulated periods (1984–2021) in the study landscape.

**Fig 3:**
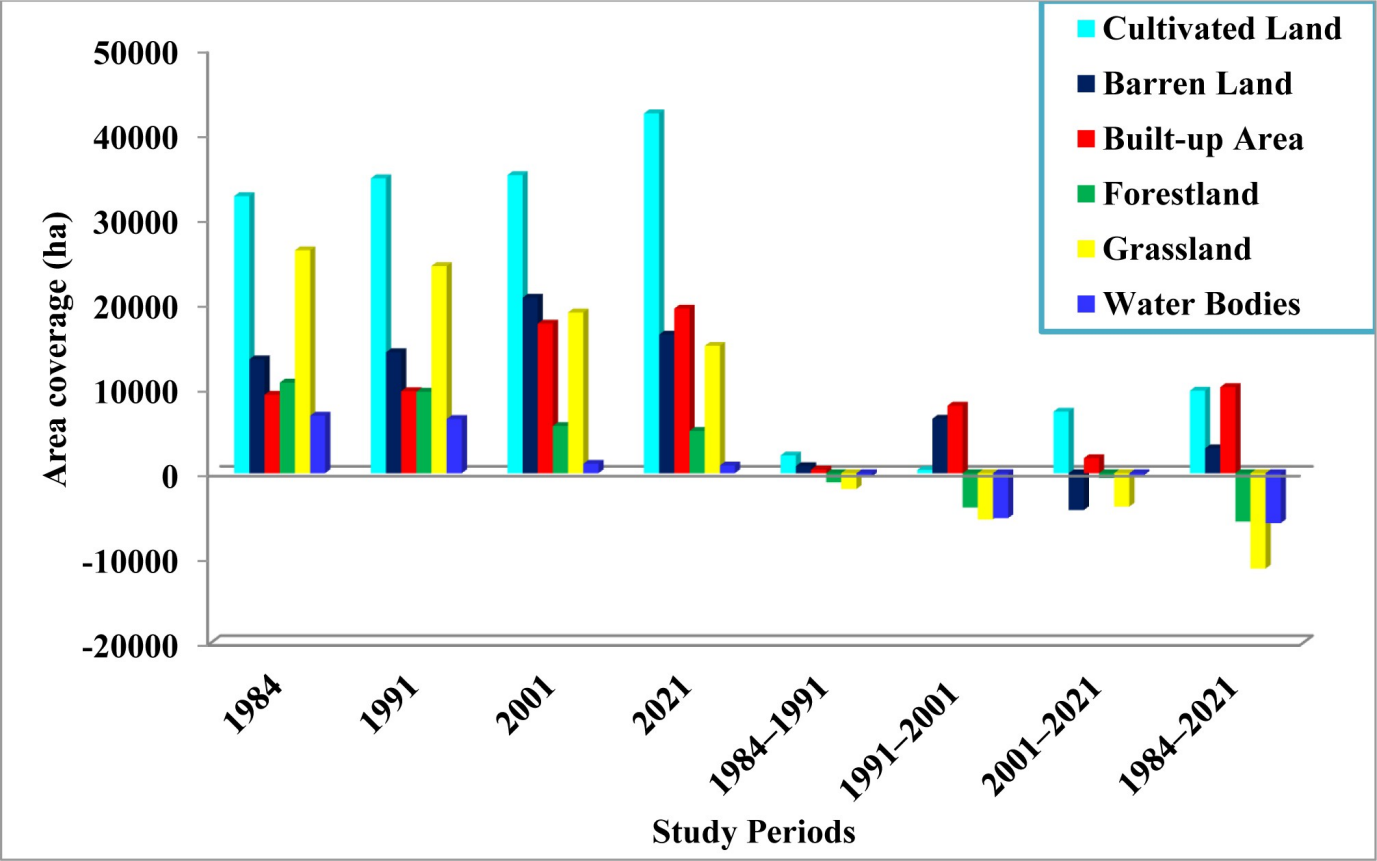
The proportion of LULC types and the area changed in hectares between 1984 and 2021. The positive changes suggest an increase/gain and the negative values indicate a decrease/loss in the status of land use types.

The LULC analysis revealed that the study areas had noteworthy changes in land use and cover during the study periods (1984–2021). The LULC change trajectories in Figs 2 and 3 represent an increasing trend in the proportion of cultivated land, built-up area, and barren land, which make up roughly 78.9%. However, the other three LULC types (forestland, grassland, and water bodies) had decreased during the study periods, together comprising 21.1% of the total (Figs 2 and 3). Notably, the proportion of forestland had decreased over the past 37 years; from 10.8% (10706.4 ha) in 1984 to 9.7% (9631.84 ha), 5.63% (5583.04 ha), and 5.0% (5005.31 ha) in 1991, 2001 and 2021, respectively, with 1.44% (-154.1 ha) of an annual rate of deforestation (Figs 2 and 3). Similar trends were also seen in the grassland, which decreased from 26.5% (26319.6 ha) in 1984 to 24.6% (24451.17 ha), 19.13% (18985.62 ha), and 15.2% (15051.85 ha) in 1991, 2001, and 2021, respectively, with an annual loss of 1.2% (Figs 2 and 3). Additionally, the area covered by water bodies declined drastically from 6.9% (6806.7 ha) in 1984 to 914.89 ha (0.9%) in 2021, at a rate of 2.34% (159.24ha) per annum (Fig 3).

**Fig 4:**
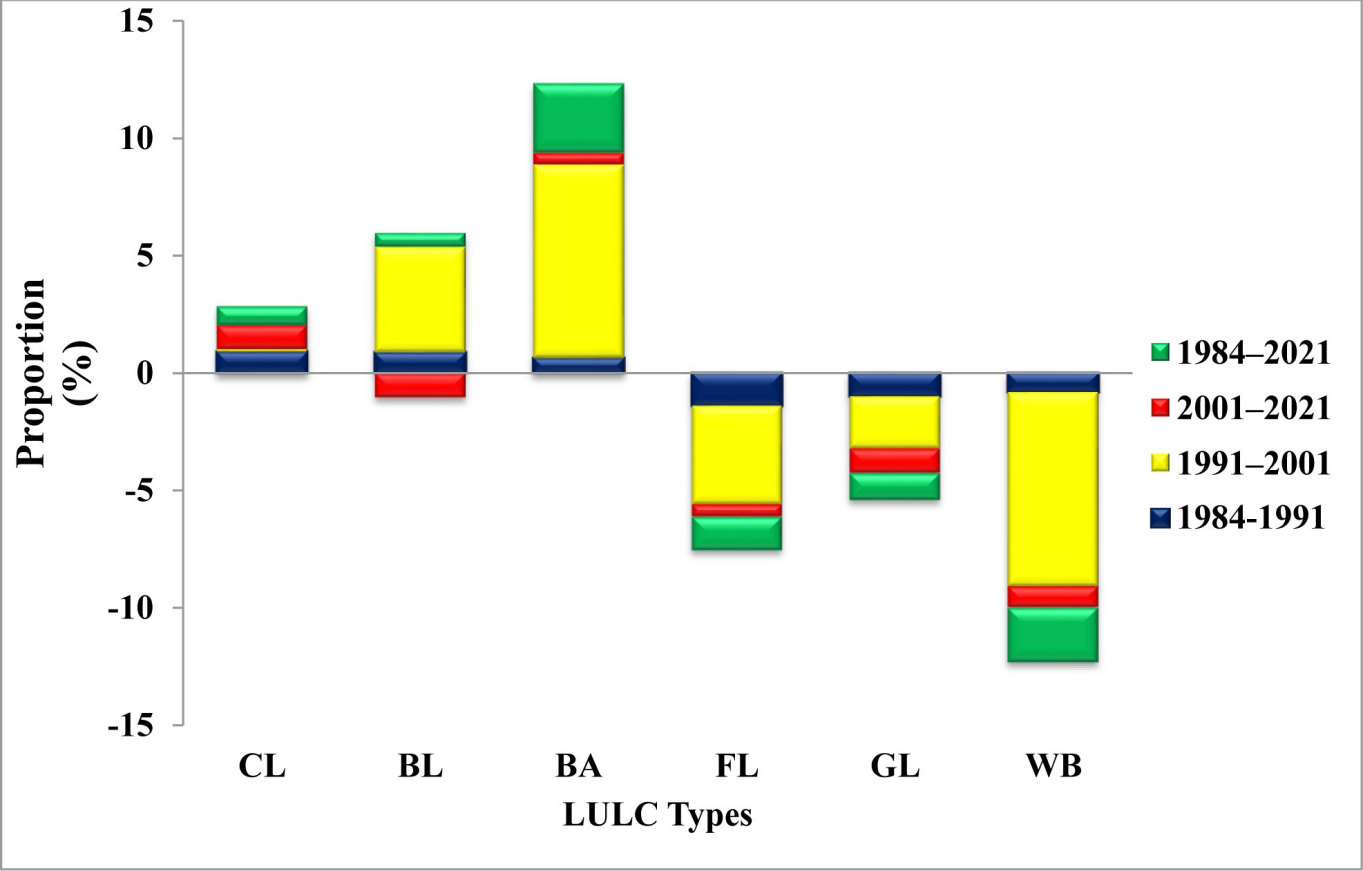
LULC changes indicating area change in percent shares between 1984 and 2021. Note: CL, Cultivated Land; BL, Barren Land; BA, Built–up Area; FL, Forest Land; GL, Grass Land; WB, Water Bodies. The positive changes suggest an increase/gain and the negative values a decrease/loss in the status of land use types.

The analysis of LULC changes showed the most significant land cover instability over the last 37 years. During the first period (1984–1991), there were changes observed in the LULC. The areas used for cultivated land, built-up, and barren land increased by 0.89% (2093.53 ha), 0.63% (1074.56 ha), and 0.87% (833.93 ha), respectively, indicating annual rates of change of 0.13%, 0.09%, and 0.12% (Figs 3 and 4). Conversely, the percentage of forest cover, grasslands, and water bodies declined during the same time frame, with an annual rate of change of -0.2% (-153.5 ha) for forestland, -0.14% (-266.9 ha) for grassland, and -0.12% (-57.3 ha) for water bodies, as depicted in Figs 3 and 4. From 1991 to 2001(second period), there was an increase in the area covered by cultivated land (384.48 ha; 0.11%), built-up area (7985.18 ha; 8.24%), and barren land (6439.77 ha; 4.5%), at a rate of 0.011%, 0.82%, and 0.45% per annum, respectively (Figs 3 and 4). At the same time frame, the percentage of forest cover, grasslands, and water bodies showed a declining trend, where the annual rates of change was -0.42% (-404.9 ha), -0.22% (-545.6 ha), and -0.83% (-529.5 ha), respectively (as used in Figs 3 and 4). Between 2001 and 2021 (third period), the proportion of cultivated and built-up area showed an incremental trend compared to the transformation of the other three LULC types that exhibited the same declining pattern as in the first and second periods (Figs 3 and 4). In contrast to the changes observed between 1991 and 2001, barren land area decreased at a rate of -1.05% (-4346.56 ha) (Fig 3). Additionally, the extent of forest area conversion was lower in the third period (2001–2021) than the first and second periods, while grassland and water bodies continued to reduce by -1.04% and -0.9%, respectively (Figs 3 and 4). The overall LULC change results (1984–2021) demonstrated the tremendous expansion of agricultural lands and settlements in the studied area, unlike the declining trend recorded for forestland, grassland, and water bodies (Fig 3).

Trends in transitions between LULC change analysis of the districts revealed that 52.98% (52,590.41 ha) of the land underwent continuous land cover conversion while 47.02% (46,667.08 ha) remained unaltered over the course of the study periods (Table 6). In particular, only 3925.7 and 8942.38 hectares of forestland and grassland remained unchanged out of 7769.2 ha and 30242.23ha in 2021, respectively (Fig 3 and Table 6). The LULC change matrix analysis in Table 6 indicated that the forestland was converted to grassland and cultivated land at proportions approximately equal to 31.5% and 12.2% over the study periods, with 0.85% and 0.33% annual rates of deforestation, respectively. In addition, the grassland was transformed into cultivated land (10432.6 ha) and built-up area (6997.4 ha) at a declining rate of 0.3% and 0.2% per annum, respectively (Table 6). In 1984, out of the total area of water bodies (6806.7 ha), 6114.4ha (89.8%) was converted to other LULC types over the study period. Of this, 15.7% and 3.3% of the water body were converted to cultivated land and built-up area, respectively (Table 6). Overall results showed that grassland experienced the greatest area coverage loss. However, we found considerably higher conversions from other LULC types to cultivated land (22479.31 ha-gains) and built-up area (18227.5 ha-gains) (Table 6).

**Table 6:** LULC transition matrix between 1984 and 2021 periods (ha).

LULC 1984–2021	BL	BA	CL	FL	GL	WB	Total (2021)	Loss
**BL**	**11887.7**	2367.2	66.4	0.65	86.99	0.57	14409.51	2521.81
**BA**	7.7	**1205.1**	9959.1	41.5	1115.96	38.4	12367.76	11162.66
**CL**	1737.5	8200.3	**20013.9**	6.99	2363.76	66.7	32389.15	12375.25
**FL**	7.6	439.2	950.91	**3925.7**	2444.25	1.54	7769.2	3843.5
**GL**	2727.2	6997.4	10432.6	1028.6	**8942.38**	114.05	30242.23	21299.85
**WB**	0.8	223.4	1070.3	0.84	92	**692.3**	2079.64	1387.34
**Total (1984)**	**16368.5**	**19432.6**	**42493.21**	**5004.28**	**15045.34**	**913.56**	**99257.49**	
**Gain**	4480.8	18227.5	22479.31	1078.58	6102.96	221.26		
**Nc**	1958.99	7064.84	10104.06	-2764.92	-15196.9	-1166.1		
**Np**	0.16	5.9	0.50	-0.70	-1.7	-1.7		

Note: BL, Barren Land; BA, Built-up Area; GL, Grassland; CL, Cultivated Land; FL, Forestland; WB, Water Bodies.

The values in each of the cells signify the amount of land converted from one land cover type to another; the bold diagonal elements indicate the area of each LULC class that remained unchanged between 1984 and 2021. The Net change (Nc) is equal to the gain minus loss and the net change–to–persistence ratio (Np) is the net change divided by diagonals of each class that did not change [30, 64, 65].

#### NDVI covers of the area, 1984–2021

**Fig 5:**
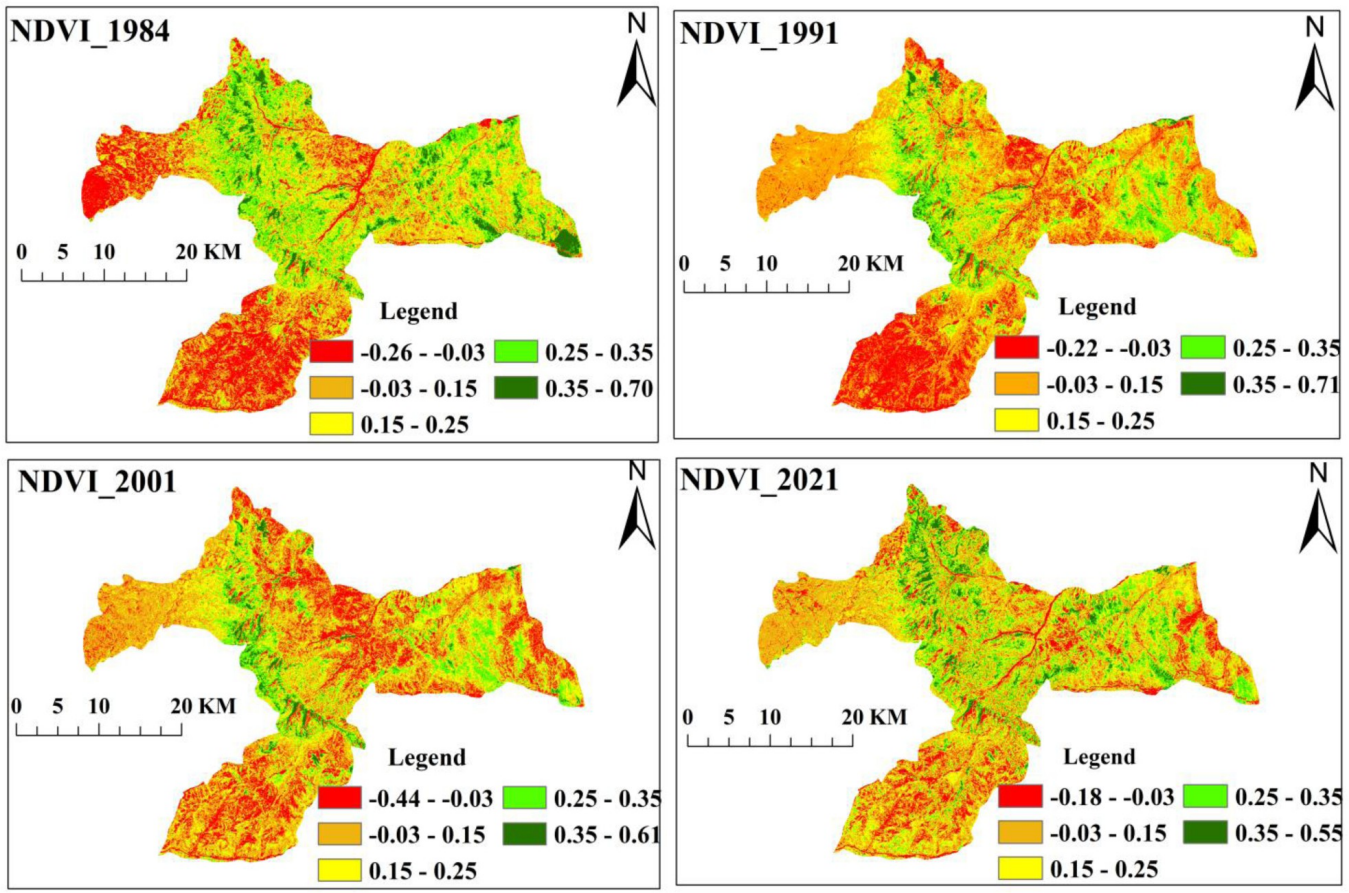
The NDVI class of the study landscape (Software: ArcGIS version 10.7.1 software, Esri, California, USA).

**Fig 6:**
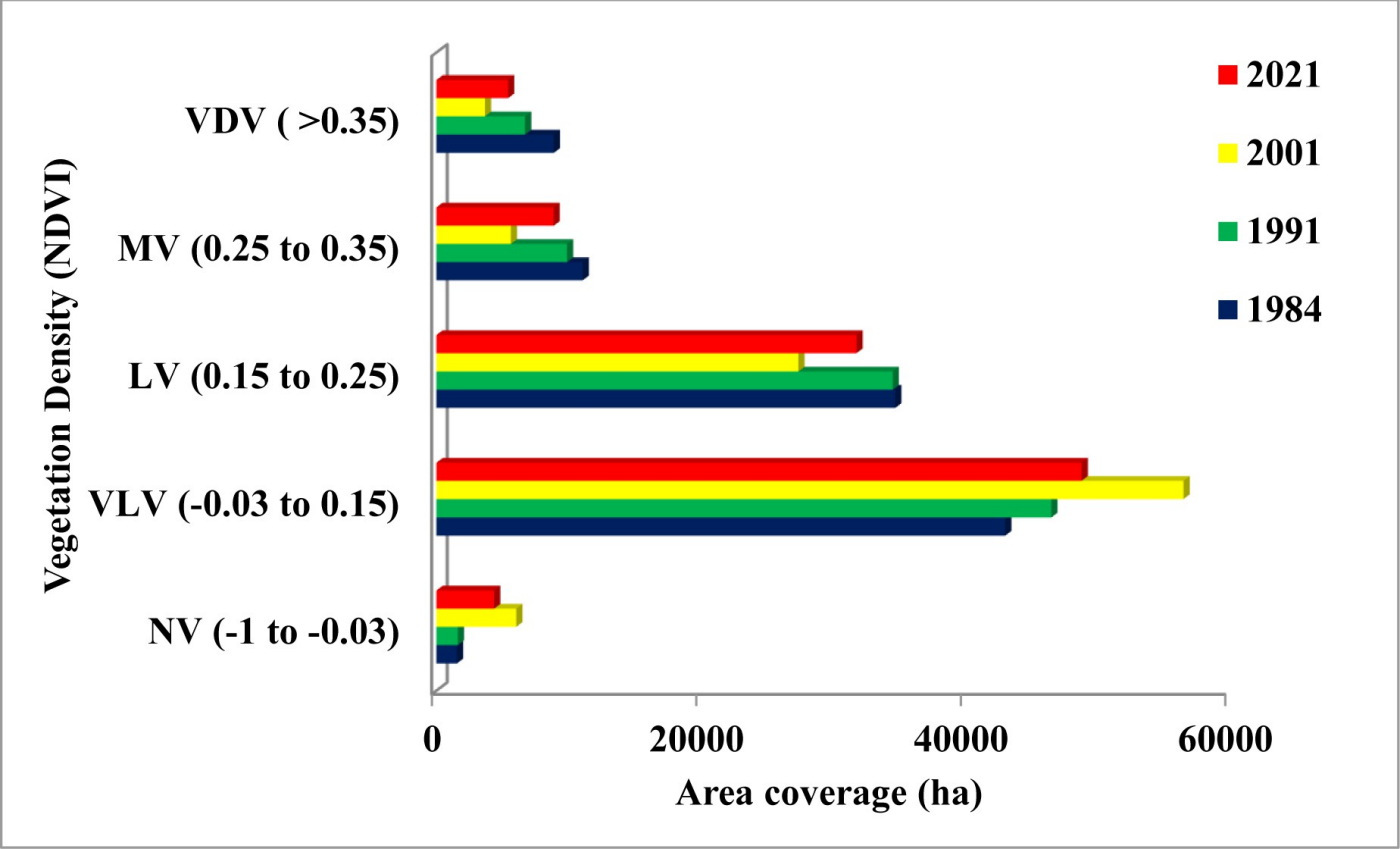
The vegetation density of the study area. Note: NV, Non–vegetation or water bodies; VLV, Very Low Vegetation; LV, Low Vegetation; MV, Moderately Vegetation; VDV, Very Dense Vegetation.

The vegetation density (NDVI) for Landsat images between 1984 and 2021 is shown in Figs 5 and 6. The findings showed that the calculated NDVI (>0.35) of the area healthy vegetation was 8.9% (8888ha) in 1984, but it dropped to 6.7% (6711.2ha) in 1991, experienced a sharp decline to 3.7% (3670.13ha) in 2001, and then noticeably increased to 5.5% (5428.8ha) in 2021. Accordingly, moderate vegetation (NDVI: 0.25 to 0.35), which covered 11.1% (11070ha), 9.96% (9891.97ha), 5.7% (5638.8ha), and 8.9% (8869.4ha) of the area in 1984, 1991, 2001, and 2021, respectively, was used to represent the grassland area. Low vegetation (NDVI: 0.15 to 0.25), an indicator of agricultural land, covered 34.96% (34785ha) and 34.8% (34507.4) of the area in 1984 and 1991, respectively. Nevertheless, the area had drastically decreased to 27.6% (27380.4ha) by 2001, and then the vegetation cover had increased to 32.0% (31780.6ha) after two decades (Figs 5 and 6). Meanwhile, very low vegetation (NDVI: -0.03 to 0.15) indicates barren land and settlement, its percentage grew from 43.3% (43030.4ha) in 1984 to 46.9% (46517.1ha), 56.9% (56517.1ha), and 49.2% (48809.7ha) in 1991, 2001, and 2021, respectively. Consistently, the non-vegetation area (NDVI: -1 to -0.03) increased from 1.5% (1564.1ha) in 1984 to 6.1% (1629.9ha) and 4.4% (4369ha) in 2001 and 2021, respectively (Figs 5 and 6).

### Spatial-temporal variation in ESV

#### Effect of LULC change on the total estimated ESVs

**Fig 7:**
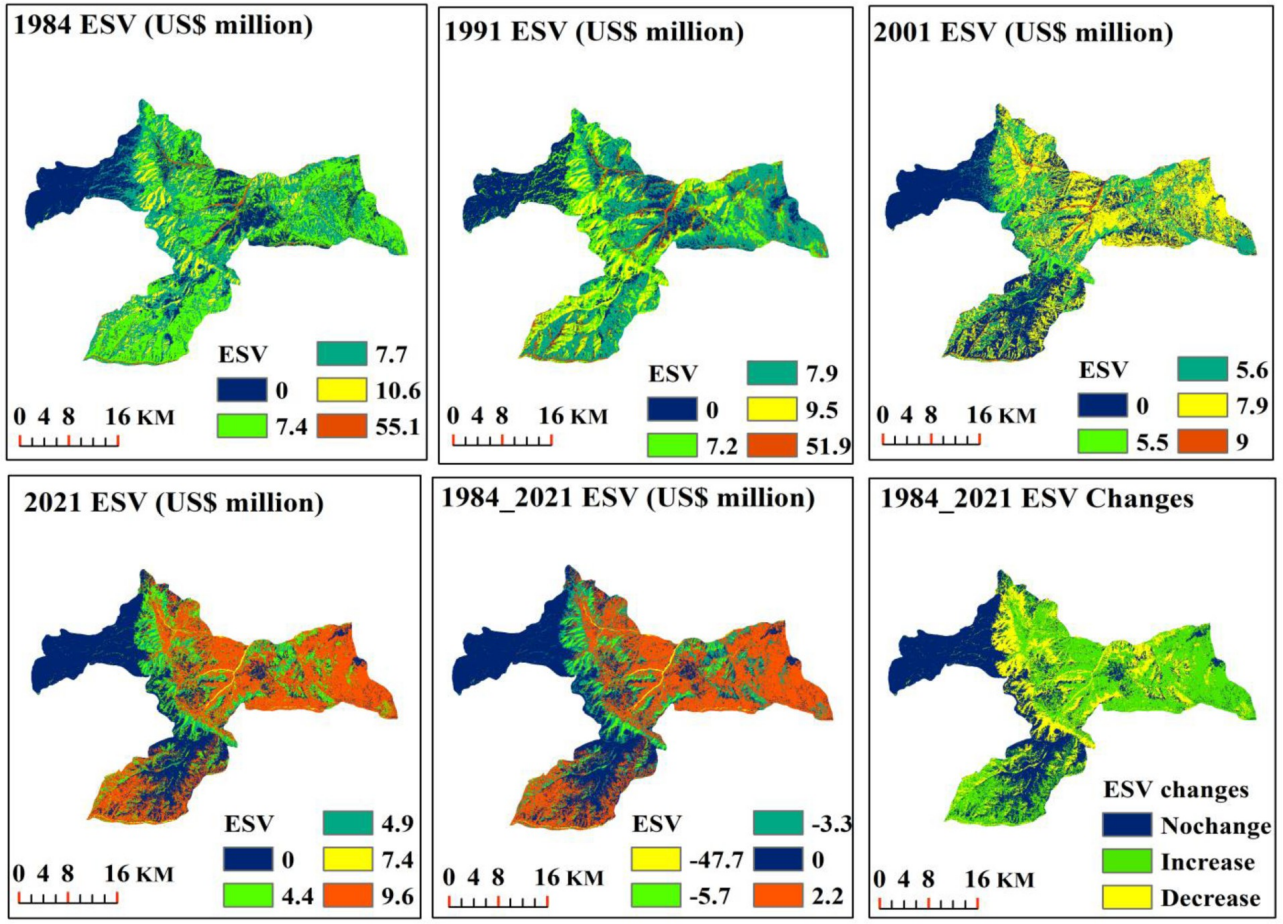
The spatial distribution of ESV across the study area.

The ecosystem service values were evaluated for the years 1984, 1991, 2001, and 2021. In 1984, the total ESV of the area was estimated to be $ 80.8 million using a modified conservative coefficient (Fig 7 and Table 7). However, the value coefficient varied by LULC type whereby, the water bodies had the highest contribution to the total ESV (55.1 million USD) during this time, followed by forestland (10.6 million USD), and grassland (7.7 million USD) (Table 7). The total ESV in 1991 was reported to be $76.4 million, in which the water bodies (51.9 million USD) were found to have the highest contribution, followed by forestland (9.5 million USD) and cultivated land. Our findings showed that the total ESV was significantly lower in the ensuing decade (2001) than in the prior research year by more than 35% (Table 7). As for each LULC type, water bodies (9.0 million USD) and cultivated land (7.9 million USD) generated the highest ESV in the area. Forestland and grasslands contributed the least to the total ESV, each with a 20% contribution (Table 7). In 2021, the total ESV was $26.4 million, with the highest contribution derived from cultivated land ($9.6 million) (Table 7). On the other hand, we considered the contribution of built-up area and bare land to the total ESV to be zero, as it was also in the modified conservative estimates (Tables 3 and 4).

**Table 7:** Ecosystem service values estimated for each land use and land cover type based on modified conservative value coefficients adopted from Kindu et al. [29].

LULC Types	ESV (US$ million)				ESV (US$ million) changes				Proportion (%) of ESV changes			
	1984	1991	2001	2021	1984–1991	1991–2001	2001–2021	1984–2021	1984–1991	1991–2001	2001–2021	1984–2021
CL	7.4	7.9	7.9	9.6	0.5	0	1.7	2.2	6.8	0	21.5	29.7
BA	-	-	-	-	-	-	-	-	-	-	-	-
FL	10.6	9.5	5.5	4.9	-1.1	-4	-0.6	-5.7	-10.4	-42.1	-10.9	-53.8
GL	7.7	7.2	5.6	4.4	-0.5	-1.6	-1.2	-3.3	-6.5	-22.2	-21.4	-42.9
BL	-	-	-	-	-	-	-	-	-	-	-	-
WB	55.1	51.9	9.0	7.4	-3.2	-42.9	-1.6	-47.7	-5.8	-82.7	-17.8	-86.6
**Total**	80.8	76.4	28.0	26.4	-4.4	-48.4	-1.6	-54.4	-5.4	-63.4	-5.7	-67.3

**Note:** CL, Cultivated Land; BL, Barren Land; BA, Built-up Area; FL, Forest Land; GL, Grass Lands; WB, Water Bodies

Over the course of the first seven years (1984–1991), the area’s total ESV decreased by 5.4% ($4.4 million). Specifically, the ESVs of water bodies, forestland, and grassland decreased by $3.2 million, $1.1 million, and $0.5million, respectively, while the ESVs of cultivated land increased by $0.5 million (Table 7). However, during the 1991–2001 period, a markedly higher conversion of forestland and water bodies to cultivated and built-up areas was observed, resulting in a total ESV loss of 48.4 USD million with a proportional loss of 63.4%. Over the same period, reductions in ESVs for the water bodies (-42.9 USD million), forestland (-4.0 USD million), and grassland (-1.6 USD million) were recorded (Table 7). In contrast to the noticeably higher ESV loss in the second period (1991–2001), the overall ESV loss between 2001 and 2021 was relatively lower (-1.6 USD million), with a proportional loss of -5.7% (Table 7). In this time frame, the total loss of ESV was mainly brought by grasslands (-21.4%) and water bodies (-17.8%). In the contrary, the total ESV of cultivated land increased by 1.7 USD million, with proportion changes of 21.5% (Table 7). Overall, from 1984–2021, the total ESV of the study landscape showed a downward trend, decreasing from 80.8 USD million to 26.4 USD million, resulting in a total decrease of 54.4 USD million (-67.3%) (Table 7). During this period, a substantial ESV loss of water bodies’ from US$ 55.1 to US$ 7.4 million (-86.6%) and terrestrial ecosystem, i.e., forestland (-53.8%) and grassland (-42.9%) were observed over the study periods, while the cultivated land exhibited an increasing pattern from US$ 7.4 to US$ 9.6 million (29.7%) (Table 7).

#### Changes in ecosystem service functions

The variations in the estimated individual ecosystem functions (ESVf) between 1984 and 2021 are shown in Table 8. Our results indicated that there was a notable variation in the rate of change in the value of ESVf during the study periods. In 1984, hydrological regulation, water supply, food production, and waste treatment contributed most to the total ESV in the north-eastern highlands of Ethiopia. They accounted for 46.02% (37.2 US$ ha^-1^yr^-1^), 17.9% (14.5 US$ ha^-1^yr^-1^), 12.1% (9.8 US$ ha^-1^yr^-1^), and 8.3% (6.7 US$ ha^-1^yr^-1^) values, respectively. However, disturbance regulation (0.07%) and cultural (0.02%) ecosystem service functions had the lowest contribution in the area (Table 8). Also, the same higher and lower ESVf contributions to the total ESV were recorded in 1991, 2001 and 2021 (Table 8). It is noteworthy that the rates of change of ESVf quite decreased from 2001 to 2021 compared to the more negative rates of change between the first (1984–1991) and second (1991 and 2001) phases (Table 8). Indeed, as a result of LULC changes, the entire ecosystem service functions showed a decreasing trend through the study periods, with the exception of food production and biological control, which lingered in change at almost the same or negligible rates across the study periods (Table 8). During the study period, the ESV function of hydrological regulation (-86.3%), water supply (-86.2%), and recreation (-81.5%) diminished drastically, much more than any other ecosystem service functions (Table 8). In the study area, therefore, expansion of agricultural land for food production was the main driver causing ESV changes in the area.

**Table 8:** The estimated value of ecosystem functions (ESVf in US$/ha/yr) within each service category across the study periods (1984–2021), overall changes, and their ESVf proportion of changes.

Ecosystem services(ES)		ESVf (US$/ha/yr)								
		1984	1991	2001	2021	Overall Change	Percentage (%) of change			
							1984–1991	1991–2001	2001–2021	1984–2021
Provisioning services	WS	14.5	13.6	2.4	2.0	-12.5	-6.2	-82.3	-16.7	-86.2
	FP	9.8	10.0	9.1	9.9	0.1	2.04	-9.0	8.8	1.02
	RM	0.55	0.5	0.3	0.3	-0.25	-9.1	-40.0	0	-45.4
	GR	0.44	0.4	0.23	0.2	-0.24	-9.1	-42.5	-13.0	-54.5
Regulatory services	Gr	0.33	0.3	0.2	0.17	-0.16	-9.1	-33.3	-15.0	-48.5
	Cr	2.4	2.1	1.3	1.1	-1.3	-12.5	-38.1	-15.4	-54.2
	Dr	0.054	0.05	0.03	0.02	-0.034	-7.4	-40	-33.3	-62.96
	Hr	37.2	35.0	6.1	5.1	-32.1	-5.9	-82.6	-16.4	-86.3
	EC	3.4	3.1	1.9	1.7	-1.7	-8.8	-38.7	-10.5	-50
	BC	1.4	1.4	1.3	1.4	0	0	-7.14	7.7	0
	WT	6.7	6.2	2.9	2.4	-4.3	-7.5	-53.2	-17.2	-64.2
Supporting services	NC	1.97	1.8	1.0	0.9	-1.07	-8.6	-44.4	-10.0	-54.3
	SF	0.13	0.1	0.07	0.06	-0.07	-23.1	-30.0	-14.3	-53.8
	Ha	0.2	0.2	0.1	0.09	-0.11	0	-50.0	-10.0	-55
	Po	1.2	1.2	1.0	1.0	-0.2	0	-16.7	0	-16.7
Recreation and culture	Re	0.54	0.5	0.1	0.1	-0.44	-7.4	-80.0	0	-81.5
	Cu	0.02	0.02	0.01	0.01	-0.01	0	-50.0	0	-50.0
**Total**		**80.83**	**76.47**	**28.04**	**26.45**		**-5.4**	**-63.3**	**-5.7**	**-67.3**

**Notes:** WS, Water Supply; FP, Food Production; RM, Raw Materials; GR, Genetic Resources; Gr, Gas regulation; Cr, Climate regulation; Dr, Disturbance regulation; Hr, Hydrological regulation; EC, Erosion Control; BC, Biological Control; WT, Waste Treatment; NS, Nutrient Cycling; SF, Soil Formation; Ha, Habitats; Po, Pollination; Re, Recreation; Cu, Cultural

#### Ecosystem coefficient of sensitivity (CS) analysis

As shown in Table 9, from 1984 to 2021 the adjusted 50% sensitivity coefficient value of each land use type ranged from 0 to 0.68, all being less than 1, which means the estimated ESVs are inelastic (low sensitive) with respect to the value of modified conservative ecosystem coefficients. Concerning each LULC type, the CS ranged from a low of 0.09–0.2 (9.1–20%) for grassland and forestland, to a high of 0.28–0.68 (28–68.7%) for water bodies and cultivated land (Table 9). However, the CS value of the water bodies was dramatically reduced from 0.68 (68.2 and 68.7%) in 1984 and 1991 to 0.32 (32%) in 2001 and 0.28 (28) in 2021 (Table 9). Meanwhile, CS for cultivated land exhibits an increment over the observed period (1984–2021). In this study, the barren land and built-up area adjusting value coefficients had no effect on ESV, and hence the sensitivity coefficient was equal to zero. Overall, the findings show that the ESVs’ estimation was accurate and practical.

**Table 9:** Percentage change in estimated total ESV and coefficient of sensitivity (CS) index resulting from an adjustment of modified conservative service value coefficients (VC).

LULC Category	1984%	CS	1991%	CS	2001%	CS	2021%	CS
Cultivated land VC±50%	9.1	0.09	10.1	0.10	28	0.28	36	0.36
Forestland VC±50%	13.1	0.13	12.1	0.12	20	0.2	19	0.19
Grassland VC±50%	9.0	0.09	9.1	0.09	20	0.2	17	0.17
Water bodies VC±50%	68.2	0.68	68.7	0.68	32	0.32	28	0.28

## Discussion

### LULC dynamics: Scenarios to the status of dry evergreen Afromontane forest

In the last 37 years, the study areas have undergone significant changes in land use and land cover, largely as a result of the conversion of forests and grassland into agricultural land and built-up area (Figs 2 and 3 and Table 6). According to the LULC analysis of changes in spatial patterns of forest loss over time, forest loss significantly increased in the second phase (1991–2001) compared to the first (1984–1991) and third (2001–2021) phases (Figs 2–6), which is primarily attributable to anthropogenic activities. Similar findings of declining forestland cover have been reported in various parts of the country [e.g., 14, 16–18, 25, 43, 66, 67]. These studies concluded that accelerated population growth and subsequent urbanization proceeded to drive uncontrollable resource utilization, and expansion of agricultural lands and settlements at the expense of forestland, which led to the LULC transitions. In addition, some researchers [e.g., 15, 17, 64, 66, 68] explained that since there was no government oversight during the interim period that followed the fall of the Derg Government of Ethiopia in 1991, illicit logging activities had occurred throughout the country. Also, this may have contributed to the decrease in forest cover in the study area during second period. However, the percentage of forest loss has gradually decreased, particularly in the third time frame from 2001 to 2021 (Figs 2 and 3). Additionally, NDVI analysis confirmed an increase in the proportion of healthy vegetation during the last two decades (Figs 5 and 6). This may be attributed to Ethiopia’s Sustainable Land Management (SLM) and Climate Resilient Green Economy (CRGE) policy initiatives enacted in 2008 and 2011, respectively [34, 69]. As part of these efforts, the government has been implementing a range of sustainable forest management intervention programs, including promoting soil and water conservation (SWC) measures (e.g., bench terraces, trenches, soil and stone bunds, etc.), planting different trees, shrubs, and herbs, establishing enclosures/exclosures, and discouraging free grazing, hence ensuring a sustainable flow of ecosystem services, as it was also reported in other similar studies [15, 65, 66]. This study also confirmed a lower annual rate of forest loss (1.44%) when compared to other studies conducted in dry evergreen Afromontane forests at various sites as a result of the active community involvement in sustainable forest management activities. For instance, Minta et al. [16], Hishe et al. [67], and Demissie et al. [68] have found out that dry evergreen forest decreased by 1.9%, 2.7%, and 1.9% between 1973 and 2015, 1995 and 2014, and 1973 and 2015 per annum, in the north west Ethiopia highlands, central Ethiopian highlands, and northern Ethiopia, respectively.

Our finding indicated that grassland was among the LULC types that changed the most rapidly during the study periods (Fig 3 and Table 6). This finding might be related to the community’s rising demand of land for farming, settlements, and grazing, as well as to institutional and policy considerations. For instance, the Amhara Regional State decreed land redeployment in 1991, allocating a large portion of the grazing lands to younger landless peasants for agriculture. There have been reports of large-scale conversion of grassland into agricultural land and settlements throughout the country [16, 44, 46, 68]. Gebrehiwot et al. [20] have also reported in the nearby agro-ecology of Ethiopia’s Abune Yosef mountain range, a decrease in shrub and grasslands and a commensurate increase in agricultural and built-up areas between 1986 and 2017. Additionally, the progressive expansion of a built-up area in the study landscape may be attributed to the rapid population growth following rapid socioeconomic transformation. According to the Central Statistical Agency [70], the study area had 164358 residents in 1994, but that number alarmingly grew to 197444 in 2007 and is projected to rise by 0.5% annually until 2021. Urbanization will undoubtedly require land for infrastructure and habitation [65], and hence the expansion may be employed at the expense of other land use types, such as natural ecosystems (grasslands, forests, and water bodies). The area’s extensive barren land is linked to the parent materials, which are of volcanic and terrestrial sedimentary rock origin [71], as well as to previous incidences of massive erosion and deforestation. This finding is consistent with Gebrehiwot et al. [20], who have reported a comparable large proportion of barren land in the surrounding district of the Abune Yosef mountain range. Similar sharp declines in the water bodies have been seen in a variety of Ethiopian landscapes. For instance, the trend of water bodies showed declines of 19.2% in western Ethiopia [25], 97.5% in the Didessa sub-basin of Ethiopia [64], and 33% in the upper Blue Nile basin of northern Ethiopia [72]. The study further showed that a substantial portion of the study landscapes experienced changes in LULC. Hence, the LULC transition matrix in Table 6 reveals the forestland, grassland, and water bodies have been highly converted into agricultural land and settlement areas. This is in line with other studies conducted in Ethiopia [e.g., 16, 17, 65], which described a marked increase in the proportion of cultivated and built-up areas that could be attributed to satisfy the demand of the expanding population for food production, shelter, and urbanization. Designing and implementing appropriate land use policies in line with the needs of settlement and agricultural land is therefore essential to bringing about unwavering regional sustainability.

### Impact of LULC dynamics on ecosystem services and functions

Globally, LULC transitions are changing rapidly, mainly owing to human-induced adversity, such as population growth, urbanization, and agricultural expansion which in turn, shrink the global ecosystem services [73, 74], and costs the world over USD 6.3 trillion, equivalent to 8.3% of global GDP in 2016 [75]. According to Costanza et al. [8], the global total terrestrial ESV decreased by 28.82%. Our estimation shows that the region’s total ESVs decreased by 32.7%, from an estimated value of 80.8 million USD in 1984 to 26.4 million USD in 2021, with an annual loss of 0.9% (Fig 7 and Table 7). This estimate is less than both the adopted local value coefficient by nearly two times and the global estimate of Costanza et al. [8]. The drastic decrease in total ESVs can be attributed to an expansion of settlements and cultivated land at the expense of natural ecosystems with high value per hectare (e.g., forestland, water bodies, and grassland). Similar losses in total ESV have been reported in various areas of Ethiopia [e.g., 29, 31–33, 65, 72] and even in some other countries like Kenya [58], China [73], Central Asia [74], Nigeria [76], and India [77], who have reported the loss of water bodies and forestland as the most LULC types contributing to the reduction in total ESV. Despite ESVs that showed a significant decline over the entire stipulated period, the proportion of ESV loss attributable to forest loss has gradually decreased over the past two decades (Fig 7 and Table 7). This is probably a result of the government’s execution of several sustainable forest management programs, which aim to restore ecosystem health in degraded areas. This is consistent with previous study reports in Ethiopia by Tesfay et al. [65], who have found that the establishment of area exclosures on degraded land has a positive effect on the ESV, with an increasing trend from 2.37 million USD in 2008 to 4.23 million USD in 2017. Importantly, the large increase in cultivated land may result in the loss of natural ecosystem services, despite appearing to be economically profitable [74]. Although several interventions and management options have been implemented in the area, the influence of cultivated land has been considerable, leading to decreases in the ESVs of forestland and grassland of 12.2% and 34.5%, respectively. Similar losses of annual ESVs due to agricultural land expansion at the expense of forestland and grasslands have been reported by Kindu et al. [29] in the Munessa-Shashemene landscape of Ethiopian highlands, Tolessa et al. [30] in the Toke Kutaye district, central highlands of Ethiopia, Solomon et al. [19] in the dry Afromontane forest of Northern Ethiopia, and Anley et al. [32] in the Rib watershed, Upper Blue Nile Basin, Ethiopia.

In response to individual ecosystem services, the results confirm that most of the ecosystem service functions have incessantly dropped over the study periods. This decline is mostly attributable to LULC changes that converted grassland, forestland, and water bodies into agricultural areas. As a result, the water supply, hydrological regulation, recreational, waste treatment, and disturbance regulation are largely affected during the stipulated periods. This could, in turn, have an impact on the resilience and resistance potential of the area to ongoing human-induced factors, as well as the livelihood of individuals who depend on the land. We compared our results with those of previous studies on the influence of LULC change on ESV in Ethiopia. And, these studies found that, despite continuous increases in the production of food, timber, and housing, human-dominated land-uses (such as urbanization and agricultural demands) have had a detrimental impact on the provision of ES [29–31, 33, 72]. This suggests understanding the drivers of changes and calling for intervention to maintain natural habitats (forestland, water bodies, and grassland) for climate regulation, hydrological regulation, water supply, water treatment, and recreation roles. In the observed periods, the inelastic ESV indicates that the results are reliable, as recommended by Li et al. [62] and Kindu et al. [29]. The highest CS value is found in water bodies because the associated value coefficient is relatively higher, while the highest value is found in cultivated land since there is more cropland in the study region than there is in other land use types.

### Limitations of the study

In this study, historical LULC changes and ESVs estimation were examined using more than three decades of Landsat imageries. However, remote sensing imageries do have some limitations. For instance, the data between 2003 and 2013 were stripped; it was extremely challenging to assess the LULC trajectories even after de-striping. We used the benefit transfer value approach to estimate the ESVs, and employed a modified conservative value coefficient adopted from Kindu et al. [29], which works based on the assumption that the value of ecosystem services would be uniform across all biomes over time. However, most ecosystems are diverse in terms of their services and functions [8, 29, 30]. Additionally, Kindu et al. [29] noted that the modified conservative value coefficient is uncertain and the ESV in settlements and barren land was not considered in this study because there was no suitable equivalent factor. However, the large-scale expansion of the built-up area in this study had an impact on the natural environments, which has the greatest ESV value coefficient. Despite these drawbacks, the assessment of spatiotemporal LULC trajectories and the estimation of ESVs in response to LULC trajectories are important in providing information for regional level decision-making and in improving the accuracy of these estimates.

## Conclusions

Our findings provide evidence that long-term human influence on the environment has resulted in a major change in LULCs in north-eastern highlands of Ethiopia during the past thirty seven years. Because of this, the built-up and agricultural areas have undergone the most changes at the expense of natural ecosystems (e.g., grassland, forestland, and water bodies). The large-scale LULC conversion may have been spurred by the accelerating socioeconomic development and rising demand for land for housing and agricultural/food production. Surprisingly, the rate of forest loss gradually decreased throughout the third study period (2001–2021), which may be the result of government efforts to manage forests sustainably. According to our research, agricultural ecosystem services increased by about 2.2 US$ million between 1984 and 2021, with a 29.7% increment per annum. Together, the total areas of forestland and water bodies dropped by -5.7 US$ million (53.8%) and -47.7 US$ million (86.6%), respectively. This substantial increase in agricultural land and decline in the natural environment may result in a decline in ecosystem services and functions (e.g., water regulation and climate regulation). Thus, policy makers must take into account the major drivers associated with the broad-scale expansion of cultivated land and built-up area and the subsequent diminution of natural ecosystems to deprive and curb further ecosystem degradation and concomitant losses of ecosystem services. As far as we are aware, evaluation of remote sensing-based analyses of land suitability and forest restoration status at a regional level and national-level are quite rare. Our findings thus pave the way for additional research into the prediction of future LULC dynamics nexus ESV using remote sensing observations, modelling land suitability analysis and forest restoration status, and the development of more accurate and trustworthy value coefficients. Moreover, using this baseline data, further studies are suggested that focus on the evaluation of the economic repercussions brought on by the LULC transition at the regional level in particular and in other geographical settings throughout the country.
